# Combinatorial Expression of *Grp* and *Neurod6* Defines Dopamine Neuron Populations with Distinct Projection Patterns and Disease Vulnerability

**DOI:** 10.1523/ENEURO.0152-18.2018

**Published:** 2018-06-13

**Authors:** Daniel J. Kramer, Davide Risso, Polina Kosillo, John Ngai, Helen S. Bateup

**Affiliations:** 1 Department of Molecular and Cell Biology, University of California, Berkeley, Berkeley, CA 94720; 2 Helen Wills Neuroscience Institute, University of California, Berkeley, Berkeley, CA 94720; 3 Division of Biostatistics and Epidemiology, Department of Healthcare Policy and Research, Weill Cornell Medicine, New York, NY 10065

**Keywords:** Dopamine Neurons, Grp, Neurod6, Retrograde Tracing, Single-Cell RNA-Sequencing, VTA, Cellular Heterogeneity

## Abstract

Midbrain dopamine neurons project to numerous targets throughout the brain to modulate various behaviors and brain states. Within this small population of neurons exists significant heterogeneity based on physiology, circuitry, and disease susceptibility. Recent studies have shown that dopamine neurons can be subdivided based on gene expression; however, the extent to which genetic markers represent functionally relevant dopaminergic subpopulations has not been fully explored. Here we performed single-cell RNA-sequencing of mouse dopamine neurons and validated studies showing that *Neurod6* and *Grp* are selective markers for dopaminergic subpopulations. Using a combination of multiplex fluorescent *in situ* hybridization, retrograde labeling, and electrophysiology in mice of both sexes, we defined the anatomy, projection targets, physiological properties, and disease vulnerability of dopamine neurons based on *Grp* and/or *Neurod6* expression. We found that the combinatorial expression of *Grp* and *Neurod6* defines dopaminergic subpopulations with unique features. *Grp^+^/Neurod6* ^+^ dopamine neurons reside in the ventromedial VTA, send projections to the medial shell of the nucleus accumbens, and have noncanonical physiological properties. *Grp^+^/Neurod6-* dopamine neurons are found in the VTA as well as in the ventromedial portion of the SNc, where they project selectively to the dorsomedial striatum. *Grp-/Neurod6* ^+^ dopamine neurons represent a smaller VTA subpopulation, which is preferentially spared in a 6-OHDA model of Parkinson’s disease. Together, our work provides detailed characterization of *Neurod6* and *Grp* expression in the midbrain and generates new insights into how these markers define functionally relevant dopaminergic subpopulations.

## Significance Statement

Recent single-cell gene profiling studies have uncovered new subpopulations of midbrain dopamine (DA) neurons defined by their specific patterns of gene expression. How these genetically defined cell types map onto known dopaminergic circuits and functionally defined cell types is unknown. This study elucidates the anatomy, circuitry, physiological properties, and disease susceptibility of midbrain DA neuron subpopulations defined by their expression of two genetic markers. This work not only advances our understanding of the dopaminergic system by providing new information about the properties of specific dopamine neuron subpopulations, it also demonstrates that unbiased genetic classification of neurons can reveal functionally relevant cell types.

## Introduction

Midbrain dopamine (DA) neurons of the substantia nigra pars compacta (SNc) and ventral tegmental area (VTA) make widespread projections throughout the brain and modulate a host of behaviors from motor function to reward learning to cognition (Björklund and Dunnett, 2007). Although they represent only ∼0.03% of neurons in the mouse brain, DA neurons are heterogeneous, as they vary significantly in their circuitry (Lammel et al., 2011; Watabe-Uchida et al., 2012; Beier et al., 2015; Menegas et al., 2015), physiology (Lammel et al., 2008; Lerner et al., 2015), gene expression (Poulin et al., 2014; La Manno et al., 2016), and response to disease (Damier et al., 1999; Di Salvio et al., 2010a; Blesa and Przedborski, 2014; Brichta and Greengard, 2014). VTA DA neurons are particularly diverse, comprising multiple subcircuits that project to different brain regions and have distinct electrophysiological properties according to their projection target (Lammel et al., 2008; Margolis et al., 2008; Morales and Margolis, 2017). Depending on their connectivity, VTA neurons can also mediate opposing behaviors, such as reward and aversion (Lammel et al., 2012), necessitating tools that can parse this functional heterogeneity to allow selective manipulation of specific VTA subpopulations (Poulin et al., 2016; Vogt Weisenhorn et al., 2016). Building on prior studies that identified genetic differences between SNc and VTA neurons (Grimm et al., 2004; Chung et al., 2005; Greene et al., 2005; Di Salvio et al., 2010b), recent single-cell gene profiling studies have uncovered further genetic heterogeneity in the DA system, including several subtypes of VTA neurons (Poulin et al., 2014; La Manno et al., 2016). However, it is currently unknown how these genetically defined classes of DA neurons map onto subtypes defined by their circuitry and physiology.

A notable feature of dopaminergic subpopulations is their differential vulnerability to disease. For example, in the neurodegenerative disorder Parkinson’s disease (PD), SNc DA neurons degenerate earlier and to a greater degree than VTA DA neurons (Damier et al., 1999; Alberico et al., 2015). The reason for this selective vulnerability is not well understood, although current hypotheses point to differences in the expression of ion channels and metabolic proteins between SNc and VTA neurons (Chung et al., 2005; Greene et al., 2005; Liss et al., 2005; Chan et al., 2007; Brichta and Greengard, 2014; Liu et al., 2014). Despite the relative sparing of the VTA compared to the SNc, 40%–77% of VTA DA neurons still degenerate (Alberico et al., 2015) and the molecular features that define susceptible versus spared VTA neurons are unknown.

Here our goal was to define dopaminergic subpopulations based on gene expression and determine how these populations map onto DA subtypes defined by physiology and circuitry. To do this, we analyzed DA neuron populations marked by two genes, *Grp* and *Neurod6,* that we identified by single-cell RNA-sequencing (RNA-seq), and which have previously been reported to mark subpopulations of VTA DA neurons (Chung et al., 2005; Greene et al., 2005; Poulin et al., 2014; La Manno et al., 2016; Viereckel et al., 2016; Khan et al., 2017). With a combination of anatomy, retrograde tracing, and physiology, we show that these genes define overlapping yet distinct DA neuron populations. We further demonstrate that the combinatorial expression of these two genes influences susceptibility to degeneration in a 6-OHDA mouse model of PD. Together, our findings further our understanding of dopaminergic cell type diversity and validate genetic approaches for defining functional cell types in the brain.

## Materials and Methods

### Mice

Animal procedures were conducted in accordance with protocols approved by the University of California, Berkeley Institutional Animal Care and Use Committee (IACUC) and Office of Laboratory Animal Care (OLAC).

For single-cell RNA-seq experiments, DAT^IRES^Cre mice (Bäckman et al., 2006; Jackson Laboratory strain #006660, RGD_12905031) were crossed and maintained with the Ai9 tdTomato Cre-reporter line (Madisen et al., 2010; Jackson Laboratory strain #007909). For physiology experiments, NEX-Cre mice were obtained from Dr. Klaus-Armin Nave (Goebbels et al., 2006) and crossed with the Ai9 mouse line. C57BL/6J mice were used for retrograde bead injections. The ages, sexes, and numbers of mice used are indicated for each experiment in the results and figure legends.

### Single-cell RNA-seq

Postnatal day (P)26 to 34 male and female DAT^IRES^Cre;Ai9 mice were briefly anesthetized with isoflurane and decapitated, and brains were removed and placed in ice-cold, oxygenated artificial CSF (ACSF; NaCl 125 mm, NaHCO_3_ 25 mm, NaH_2_PO_4_ 1.25 mm, KCl 2.5 m, MgCl_2_ 1 mm, CaCl_2_ 2 mm, glucose 25 mm). The brain was cut coronally into 275-µm sections on a vibratome (Leica VT1000 S) in oxygenated ice-cold choline cutting solution (choline chloride 100 mm, NaHCO_3_ 25 mm, NaH_2_PO_4_ 1.25 mm, KCl 2.5 mm, MgCl_2_ 7 mm, CaCl_2_ 0.5 mm, glucose 25 mm, sodium ascorbate 11.6 mm, sodium pyruvate 3.1 mm). Midbrain sections were incubated for 15 min in ACSF at 34˚C. Midbrain (including the hypothalamus) was dissected in ACSF using forceps under a dissection microscope. Midbrain sections were incubated in 10 ml oxygenated papain solution (papain 10 U/ml (Worthington #LK003176) in ACSF with 10 mm HEPES, 10 U/ml DNase, 2.5 mm EDTA, 2.5 mm cysteine, 1 mm kynurenic acid, and 5 mm CaCl_2_) for 25 min at 34˚C. Following papain digestion, tissue was placed into 9 ml oxygenated STOP-ovomucoid inhibitor solution [1 ml/mg ovomucoid (Worthington #LK003182) in HEPES-ACSF, 10 U/ml DNase, 1 mm kynurenic acid, and 5 mm CaCl_2_] and bubbled gently at 34˚C. 8 ml of the supernatant solution was removed, and the tissue was triturated serially in the remaining 2 ml of solution with polished 3-, 2-, and 1-mm glass pipettes to create a single-cell suspension. The 2 ml single-cell suspension was spun down in a 20% Percoll solution (600 µl Percoll (Sigma #P4937) in 2.4 ml STOP-ovomucoid solution at 430 × *g* for 6 min at room temperature (RT). The supernatant was aspirated, leaving ∼50 µl of solution and the cell pellet. The pellet was resuspended in 1 ml HEPES-ACSF with 1 mm kynurenic acid and 5% FBS (Life Technologies #16140063).

This single-cell suspension was sorted on a BD Influx cell sorter in the Flow Cytometry Facility at UC Berkeley. Cells were gated for PI-/tdTomato^+^ and sorted into a PCR tube. Based on the number of neurons sorted and cell viability count, neurons were brought to ∼200,000 neurons/ml. Neurons were then put into a large Fluidigm C1 chip, and each of the 96 wells was visually inspected to verify cell presence, cell health, and tdTomato expression. Wells containing cell doublets were excluded from further processing.

Cells went through single-cell mRNA extraction using the Fluidigm C1 system in the Functional Genomics Laboratory at UC Berkeley. Single-cell cDNA was removed and measured via Qubit. Any cell that gave <0.3 µg/ml of cDNA was removed due to likely low quality. cDNA from single cells that passed the initial quality check was diluted to 0.3 µg/ml. 379 single-neuron cDNA extracts were library prepped using the Nextera DNA library prep protocol (Illumina #FC-121-1012). The cDNA was then sequenced on a HiSeq 2500 in the Vincent J. Coates Genomics Sequencing Laboratory at UC Berkeley.

### Single-cell RNA-seq preprocessing

Reads were aligned to the GRCm38.3 (mm10, patch release 3) mouse genome assembly with *Tophat2* (v. 2.1.1; Kim et al., 2013), and low-quality reads were removed with *Trimmomatic* (v. 0.3.2; Bolger et al., 2014). Gene expression was quantified using *featureCounts* (v. 1.5.0-p3; Liao et al., 2014) and RefSeq transcript annotation. Reads that aligned to more than one gene as well as chimeric fragments were excluded. We used a quality control (QC) pipeline that computes an extensive set of quality metrics, relying in part on *FastQC* (v. 0.3.2) and the *Picard tools* (v. 2.5.0 with *samtools* 1.3.1) as done previously (Fletcher et al., 2017). We used the Bioconductor package *scone* (https://bioconductor.org/packages/scone; v. 0.99.6) to perform data-adaptive cell and gene filtering. This yielded the following exclusion criteria: any cell with fewer than 500,000 aligned reads or a percentage of aligned reads below 85%. In addition, we filtered out cells with large dropout rates, as defined by the “false-negative curves” of *scone*. This procedure resulted in a total of 232 retained cells (out of 379). Finally, we retained only those genes having at least 10 reads in at least 10 cells (10,983 genes).

### Single-cell RNA-seq statistical analysis

We performed and assessed several normalization schemes using *scone* (Cole et al., 2017) and selected full-quantile normalization (Bolstad et al., 2003; Bullard et al., 2010). We then applied principal component analysis on the normalized data and retained the first 50 principal components, which explained 41% of the variance. Cluster analysis was performed on the first 50 principal components using the *RSEC* method (Risso et al., 2018b) implemented in the Bioconductor package *clusterExperiment* (https://bioconductor.org/packages/clusterExperiment; v. 1.0.0), as previously described (Fletcher et al., 2017). We used RSEC with the following specific parameters: alphas = 0.3, minSizes = 5, combineProportions = 0.5; all other parameters were left at their default values. RSEC found 9 stable clusters. We used *limma* (v. 3.30.13) with *voom* correction weights (Law et al., 2014), as implemented in the *clusterExperiment* function *getBestFeature*, to find marker genes for each cluster. To visualize the clustering results, we applied ZINB-WaVE (Risso et al., 2018a; with K = 10) for dimensionality reduction, followed by t-distributed stochastic neighbor embedding (t-SNE; perplexity parameter set to 20; van der Maaten and Hinton, 2008).

### Accession number and code accessibility

The accession number for the RNA-seq data reported in this paper is GEO: GSE115070. The computer code for the single-cell RNA-seq analysis is available at https://github.com/drisso/striatum. This code was run on an Apple Mac computer with macOS Sierra 10.12.4 operating system.

### Fluorescent *in situ* hybridization

To visualize mRNA we used the RNAScope fluorescent *in situ* hybridization (FISH) method (ACDBio). Fresh-frozen tissue was processed as per RNAScope instructions. Briefly, mouse brain tissue from male and female mice aged P21–P120 was fresh-frozen in OCT on dry ice and stored at least overnight at –80˚C. Tissue was then cut on a cryostat (Leica Microm HM550) into 12 µm sections and mounted onto slides. Slides were fixed in 4% PFA in 1× PBS for 15 min. Slides were dehydrated using 5 min incubations in 50%, 70%, and twice with 100% ethanol. Slides were incubated with RNAScope Protease IV (ACDBio #322340) at RT for 30 min and washed with 1× PBS. FISH was then performed using the RNAScope Multiplex Fluorescent assay (ACDBio #320850) per the manufacturer’s instructions and protocols. Following FISH, slides were coverslipped using ProLong Gold Antifade mounting media (Invitrogen #P36934). RNAScope probes used: mM-Neurod6 (#444851), mM-Grp (#317861), mM-Th (#317621), mM-Slc6a3 (#315441), mM-Lypd1 (#447081).

### Microscopy and image analysis

Two confocal microscopes were used to take *Z*-stack images of FISH-labeled or immunostained sections: an Olympus FV1000 with a 20× Nikon objective and a Zeiss LSM 710 AxioObserver with Zeiss 10×, 20×, and 63× objectives housed in the Molecular Imaging Center at UC Berkeley. Images were analyzed using the Fiji image analysis toolbox. Cells were considered positive for *Neurod6*, *Grp*, or *Lypd1* if they contained three fluorescent puncta within the boundary created by a cell marker: *Slc6a3* (DAT), *Th*, or DAPI.

### Retrobead and virus intracranial injections

P14–P18 wild-type male and female mice were used for retrograde labeling experiments. Green Retrobeads IX (Lumaflour #G180) were diluted 1:7 in sterile 1× PBS unless otherwise noted. Beads were bilaterally injected using a pulled glass pipette. The following coordinates from bregma and bead volumes were used to target each projection site: dorsomedial striatum (DMS; M/L ±1.35 mm, A/P +0.75 mm, D/V –2.60 mm, 400 nl beads), dorsolateral striatum (DLS; M/L ±2.15 mm, A/P +0.70 mm, D/V –2.50 mm, 400 nl beads), nucleus accumbens (NAc) medial shell (M/L ±0.75 mm, A/P +1.20 mm, D/V –4.15 mm, 300 nl beads), NAc core (M/L +/1.2 mm, A/P +1.10 mm, D/V –4.05 mm, 300 nl beads), NAc lateral shell (M/L ±1.75 mm, A/P +1.05 mm, D/V –3.95 mm, 300 nl beads), basolateral amygdala (M/L ±2.65 mm, A/P –1.05 mm, D/V –4.40 mm, 120 nl beads at 1:3 dilution), medial prefrontal cortex (four injections per hemisphere at two different depths per injection: M/L ±0.35 mm, A/P +1.50 mm, +1.65 mm, +1.80 mm, and +1.95 mm, D/V –2.00 mm and –1.40 mm, 400 nl total per hemisphere), and lateral septum (M/L ±0.45 mm, A/P +0.40 mm, D/V –2.70 mm, 300 nl beads).

To allow for sufficient DA neuron labeling, mice were sacrificed at various time points following injection: 7 d for the DMS and DLS, 21 d for the prefrontal cortex, and 14 d for the NAc (medial shell, core, and lateral shell), lateral septum and amygdala. Brains were harvested and cut to separate the injection site and midbrain. The midbrain was frozen for cryostat sectioning as described above for FISH. The brain region containing the injection site was incubated in 4% PFA overnight at 4˚C, then cryoprotected in 30% sucrose in 1× PB until the tissue sank. Injection site tissue was sectioned on a freezing microtome, mounted with Vectashield hardset mounting media with DAPI (Vector laboratories #H-1500), and analyzed for bead expression at the injection site. Brains with correctly targeted injection sites and minimal off-target bead expression were chosen for analysis.

To selectively label NEX-Cre–expressing neurons in the VTA, we unilaterally injected 800 nl of a Cre-dependent tdTomato virus (AAV1.CAG.Flex.tdTomato.WPRE.bGH, Penn Vector Core #AV-1-ALL864) into heterozygous NEX-Cre mice at P16. To target the VTA, we used the following coordinates from bregma: M/L ±0.25 mm, A/P –2.9 mm, D/V –4.45 mm.

### Immunohistochemistry

Immunohistochemistry was performed as described previously (Bateup et al., 2013). The following antibodies were used: tyrosine hydroxylase (TH, ImmunoStar #22941, RRID:AB_572268), Alexa Fluor 488 goat anti-mouse secondary (Thermo Fisher Scientific #A-11001, RRID:AB_2534069), Alexa Fluor 633 goat anti-mouse secondary (Thermo Fisher Scientific #A-21050, RRID:AB_2535718), streptavidin Alexa Fluor 488 conjugate (Thermo Fisher Scientific #S32354, RRID:AB_2315383), and streptavidin Alexa Fluor 633 conjugate (Thermo Fisher Scientific #S21375, RRID:AB_2313500).

### Electrophysiology

275-µm-thick coronal midbrain slices were prepared from P56–P105 NEX-Cre;Ai9 or DAT^IRES^Cre;Ai9 mice of both sexes on a vibratome (Leica VT1000 S) in ice-cold high Mg^2+^ ACSF containing, in mm: 85 NaCl, 25 NaHCO_3_, 2.5 KCl, 1.25 NaH_2_PO_4_, 0.5 CaCl_2_, 7 MgCl_2_, 10 glucose, and 65 sucrose. Slices were recovered for 15 min at 34°C followed by 50 min at RT in ACSF containing, in mm: 130 NaCl, 25 NaHCO_3_, 2.5 KCl, 1.25 NaH_2_PO_4_, 2 CaCl_2_, 2 MgCl_2_, and 10 glucose. NEX-Cre^+^ VTA neurons were identified by tdTomato fluorescence in NEX-Cre;Ai9 mice. SNc neurons were defined either by the presence of green retrobeads injected into the dorsolateral striatum in NEX-Cre;Ai9 mice or by tdTomato fluorescence and anatomic location in DAT^IRES^Cre;Ai9 mice. For whole-cell recordings, 2.5–6 mΩ glass pipettes were filled with a potassium-based internal solution containing, in mm: 135 KMeSO_3_, 5 KCl, 5 HEPES, 4 Mg-ATP, 0.3 Na-GTP, 10 phospho-creatine, 1 EGTA, and 4 mg/ml neurobiotin (Vector laboratories #SP-1120). Recordings were obtained using a MultiClamp 700B amplifier (Molecular Devices) and ScanImage software. Passive membrane properties were recorded in voltage clamp with the membrane held at –70 mV. Spontaneous action potentials were recorded in current clamp. In current clamp, 500 ms steps of negative current were delivered (–25 to –150 pA) to hyperpolarize the membrane to approximately –100 mV. During the steps, current was injected to maintain the baseline membrane potential at –70 mV. All recordings were performed at RT in the presence of synaptic blockers (NBQX 10 µm, CPP 10 µm, picrotoxin 50 µm, final concentration). Data were analyzed in Igor (Wavemetrics) using custom scripts.

### 6-OHDA injection

6-hydroxydopamine (6-OHDA; 6-hydroxydopamine hydrobromide with ascorbic acid: Sigma-Aldrich #H116-5MG) injections were made into the medial forebrain bundle of P120 female mice as previously described (Thiele et al., 2012). 30 min before 6-OHDA injection, a solution containing 0.5 mg/ml pargyline (Sigma Aldrich #P8013) and 2.5 mg/ml desipramine hydrochloride (Tocris #3067) was injected i.p. at a dose of 5 mg/kg pargyline and 25 mg/kg desipramine. 200 nl of freshly prepared 15 mg/ml 6-OHDA in sterile saline + 0.02% ascorbic acid were injected into the medial forebrain bundle (MFB, coordinates from bregma: M/L ± 1.2 mm, A/P 1.2 mm, D/V –4.90 mm). Adult female wild-type mice were used as younger mice and male mice showed poor recovery following injection. 250–350 µl of meloxicam (5–10 mg/kg dose) was injected subcutaneously as an analgesic.

Mice were monitored daily following the injection to ensure recovery. Kitten Milk Replacement (Santa Cruz #sc-362120) was fed to mice daily for up to 2 wk following the injection to aid recovery and meloxicam was injected subcutaneously to alleviate pain if necessary. Motor function was assessed using the cylinder test each week following the injection (see below). 4 wk following 6-OHDA injection, mice were quickly anesthetized using isoflurane and decapitated, and their brains were fresh-frozen as described above for FISH.

### Cylinder test

To test the severity of Parkinsonian-like symptoms following unilateral 6-OHDA injection, we used the cylinder test to score limb use asymmetry. Mice were habituated to the behavior room for a minimum of 30 min during their dark cycle under red light illumination. Mice were placed into a clear plastic cylinder 12 cm in diameter and 20 cm in height. The cylinder was placed next to two mirrors to visualize paw use. The mouse was both video recorded and observed by the experimenter while it was allowed to move around freely in the cylinder for 10 min. Full 360° ipsiversive and contraversive rotations (relative to the 6-OHDA injection side) were counted. Forelimb asymmetry was measured by counting the number of times the ipsilateral paw, contralateral paw, or both paws were used for support when the mouse reared against the wall of the cylinder. The percentage of ipsilateral or contralateral paw use was calculated based on total rears (e.g., ipsilateral paw touches/ipsilateral + contralateral + both paw touches). A greater number of ipsiversive turns and ipsilateral paw use indicates a successful 6-OHDA injection and unilateral Parkinsonian-like symptoms.

### Experimental design and statistical analysis

A one-way ANOVA was used to compare the means of three or more groups. *Post hoc* pairwise comparisons were made using either Bonferroni’s or Tukey’s multiple comparisons tests. Unpaired or paired two-tailed *t* tests were used to compare the means of two groups. A paired, one-way ANOVA with Dunnett’s multiple comparisons *post hoc* test was used to compare DA neuron subpopulations to the entire VTA DA population for the 6-OHDA experiments. Data are reported as the mean ± SEM. Superscript letters listed with *p*-values correspond to the statistical tests shown in Table 1. *p*-values were corrected for multiple comparisons.

**Table 1.** Statistical analysis

Location	Data structure	Type of test	CI/power	*P* value	Comparison
a	Normally distributed	One-way ANOVA	0.97	<0.0001	*Grp* expression in midbrain subregions
	Normally distributed	Tukey’s *post hoc*	6.508 to 17.74	<0.0001	*Grp* IF vs. PN/PIF
	Normally distributed	Tukey’s *post hoc*	34.77 to 46	<0.0001	*Grp* IF vs. PBP
	Normally distributed	Tukey’s *post hoc*	35.1 to 46.33	<0.0001	*Grp* IF vs. SNc-V
	Normally distributed	Tukey’s *post hoc*	57.26 to 68.49	<0.0001	*Grp* IF vs. SNc-D
	Normally distributed	Tukey’s *post hoc*	22.65 to 33.88	<0.0001	*Grp* PN/PIF vs. PBP
	Normally distributed	Tukey’s *post hoc*	22.97 to 34.2	<0.0001	*Grp* PN/PIF vs. SNc-V
	Normally distributed	Tukey’s *post hoc*	45.13 to 56.37	<0.0001	*Grp* PN/PIF vs. SNc-D
	Normally distributed	Tukey’s *post hoc*	–5.292 to 5.942	0.9998	*Grp* PBP vs. SNc-V
	Normally distributed	Tukey’s *post hoc*	16.87 to 28.1	<0.0001	*Grp* PBP vs. SNc-D
	Normally distributed	Tukey’s *post hoc*	16.55 to 27.78	<0.0001	*Grp* SNc-V vs. SNcD
b	Normally distributed	One-way ANOVA	0.96	<0.0001	*Neurod6* expression in midbrain subregions
	Normally distributed	Tukey’s *post hoc*	–0.4177 to 9.918	0.0842	*Neurod6* IF vs. PN/PIF
	Normally distributed	Tukey’s *post hoc*	16.28 to 26.62	<0.0001	*Neurod6* IF vs. PBP
	Normally distributed	Tukey’s *post hoc*	34.69 to 45.03	<0.0001	*Neurod6* IF vs. SNc-V
	Normally distributed	Tukey’s *post hoc*	35.82 to 46.16	<0.0001	*Neurod6* IF vs. SNc-D
	Normally distributed	Tukey’s *post hoc*	11.53 to 21.87	<0.0001	*Neurod6* PN/PIF vs.
	Normally distributed	Tukey’s *post hoc*			PBP
	Normally distributed	Tukey’s *post hoc*	29.94 to 40.28	<0.0001	*Neurod6* PN/PIF vs. SNc-V
	Normally distributed	Tukey’s *post hoc*	31.07 to 41.41	<0.0001	*Neurod6* PN/PIF vs. SNc-D
	Normally distributed	Tukey’s *post hoc*	13.24 to 23.58	<0.0001	*Neurod6* PBP vs. SNc-V
	Normally distributed	Tukey’s *post hoc*	14.37 to 24.71	<0.0001	*Neurod6* PBP vs. SNc-D
	Normally distributed	Tukey’s *post hoc*	–4.043 to 6.293	0.9699	*Neurod6* SNc-V vs. SNc-D
c	Normally distributed	One-way ANOVA	0.522	0.036	*Grp* expression by age
	Normally distributed	Tukey’s *post hoc*	–1.112 to 2.462	0.5636	*Grp* 2 wk vs. 8 wk
	Normally distributed	Tukey’s *post hoc*	–3.087 to 0.4872	0.1603	*Grp* 2 wk vs. 16 wk
	Normally distributed	Tukey’s *post hoc*	–3.762 to – 0.1878	0.0316	*Grp* 8 wk vs. 16 wk
d	Normally distributed	One-way ANOVA	0.56	0.0236	*Neurod6* expression by age
	Normally distributed	Tukey’s *post hoc*	–2.859 to 1.109	0.4655	*Neurod6* 2 wk vs. 8 wk
	Normally distributed	Tukey’s *post hoc*	–4.384 to – 0.4162	0.0201	*Neurod6* 2 wk vs. 16 wk
	Normally distributed	Tukey’s *post hoc*	–3.509 to 0.4588	0.1351	*Neurod6* 8 wk vs. 16 wk
e	Normally distributed	Unpaired *t* test	–7.68 to 3.147	0.3728	*Grp* IF M vs. F
f	Normally distributed	Unpaired *t* test	–12.78 to 0.4155	0.0634	*Grp* PN/PIF M vs. F
g	Normally distributed	Unpaired *t* test	–1.924 to 4.89	0.3549	*Grp* PBP M vs. F
h	Normally distributed	Unpaired *t* test	–7.321 to 5.188	0.7119	*Grp* SNCv M vs. F
i	Normally distributed	Unpaired *t* test	–0.6657 to 1.199	0.5383	*Grp* SNCd M vs. F
j	Normally distributed	Unpaired *t* test	–13.48 to 4.412	0.2852	*Neurod6* IF M vs. F
k	Normally distributed	Unpaired *t* test	–8.615 to 3.682	0.3924	*Neurod6* PN/PIF M vs. F
l	Normally distributed	Unpaired *t* test	–1.028 to 2.095	0.4642	*Neurod6* PBP M vs. F
m	Normally distributed	Unpaired *t* test	–2.251 to 0.1514	0.0801	*Neurod6* SNCv M vs. F
n	Negative binomial	Likelihood ratio test	n/a	0.5113	*Neurod6* M vs. F RNASeq data
o	Negative binomial	Likelihood ratio test	n/a	0.6835	*Grp* M vs. F RNASeq data
p	Normally distributed	Unpaired *t* test	–9.156 to –1.532	0.0075	Vm – SNc vs. NEXCre
q	Normally distributed	Unpaired *t* test	–1222 to –729.4	<0.0001	Rm – SNc vs. NEXCre
r	Normally distributed	Unpaired *t* test	23.67 to 43.99	<0.0001	Cm – SNc vs. NEXCre
s	Normally distributed	Unpaired *t* test	11.3 to 21.88	<0.0001	AP height – SNc vs. NEX-Cre
t	Normally distributed	Unpaired *t* test	3.372 to 9.524	0.0002	AHP – SNc vs. NEXCre
u	Normally distributed	Unpaired *t* test	6.564 to 10.49	<0.0001	Sag – SNc vs. NEXCre
v	Normally distributed	Unpaired *t* test	4.295 to 7.773	<0.0001	Rebound – SNc vs. NEX-Cre
w	Normally distributed	One-way ANOVA	0.85	0.0002	Cylinder test
	Normally distributed	Tukey’s *post hoc*	6.33 to 55.07	0.0163	Saline vs. 6-OHDA paw
	Normally distributed	Tukey’s *post hoc*	–55.27 to –6.53	0.0157	Saline vs. both paws
	Normally distributed	Tukey’s *post hoc*	–85.97 to –37.23	0.0002	6-OHDA vs. both paws
x	Normally distributed	Paired one-way ANOVA	0.96	<0.0001	Cell population survival
	Normally distributed	Dunnett’s *post hoc*	–69.31 to –34.69	0.0022	All *TH^*+*^* vs. *Grp*-/ *Neurod6* ^+^
	Normally distributed	Dunnett’s *post hoc*	–6.01 to 15.16	0.3169	All *TH^*+*^* vs. *Grp* ^+^/ *Neurod6* ^+^
	Normally distributed	Dunnett’s *post hoc*	2.07 to 15.78	0.0246	All *TH^*+*^* vs. *Grp^*+*^/ Neurod6*-
	Normally distributed	Dunnett’s *post hoc*	0.6149 to 15.14	0.0403	All *TH^*+*^* vs. *Lypd1* ^+^/*Neurod6*-
y	Normally distributed	Paired *t* test	–3.593 to 3.043	0.8091	*Lypd1^*+*^/Neurod6* ^+^ saline vs. 6-OHDA

## Results

### Single-cell RNA-seq of midbrain dopamine neurons

**Figure 1.**
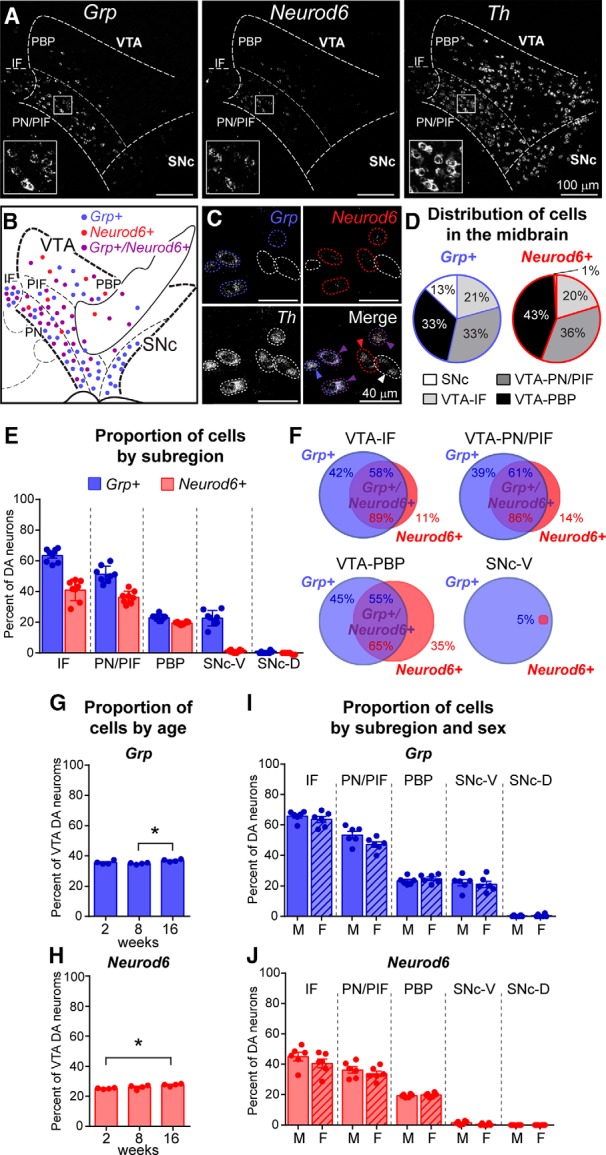
Anatomic analysis of *Grp* and *Neurod6*-expressing DA neurons in the midbrain. ***A***, Confocal images of multiplex fluorescent *in situ* hybridization using probes against *Grp* (left), *Neurod6* (middle), and tyrosine hydroxylase (*Th*, right) mRNA. Inset boxes show higher-magnification images of the boxed regions. ***B***, Schematic showing the location of *Grp* ^+^ (blue), *Neurod6^+^* (red), and *Grp^+^/Neurod6^+^* (purple) DA neurons in the midbrain. ***C***, High-magnification confocal images showing *Th*-positive VTA neurons expressing *Grp* (blue circles), *Neurod6* (red circles), *Grp* and *Neurod6* (purple circles), or neither marker (white circles). ***D***, Charts show the distribution of *Grp^+^* or *Neurod6* ^+^ neurons across different midbrain regions, expressed as a percentage of the total *Grp^+^* or *Neurod6* ^+^ population. Only DA neurons, defined by expression of *Th* mRNA, were included in the analysis, *n* = 4377 cells quantified from 8 mice (4 male and 4 female). ***E***, Quantification of the number of *Th* ^+^ DA neurons that coexpress *Grp* or *Neurod6* in subregions of the VTA and SNc. Bars represent mean ± SEM, dots represent the values from individual mice, *n* = 4377 *Th* ^+^ cells quantified from 4 male and 4 female mice. *Grp* one-way ANOVA, *p* < 0.0001; Tukey’s *post hoc* tests revealed significant (*p* < 0.0001) differences for each subpopulation compared to all others except the PBP versus SNc-V (*p* = 0.9998). *Neurod6* one-way ANOVA, *p* < 0.0001; Tukey’s *post hoc* tests revealed significant (*p* < 0.0001) differences for each subpopulation compared to all others except the IF versus PN/PIF (*p* = 0.0842) and SNc-V versus SNc-D (*p* = 0.9699). ***F***, Venn diagrams display the extent of overlap between the *Grp* and *Neurod6*-expressing DA neuron populations across four midbrain subregions. 1833 cells were quantified from 8 mice (4 male and 4 female). ***G***, ***H***, Graphs display the mean ± SEM percentage of *Grp^+^* (***G***) or *Neurod6* ^+^ (***H***) DA neurons in the VTA at 2, 8, and 16 wk. Statistical comparisons were made with a one-way ANOVA with Tukey’s *post hoc* test; *Grp* 8 wk (34.9%) vs. 16 wk (36.9%), *, *p* = 0.0316, *Neurod6* 2 wk (25.2%) vs. 16 wk (27.6%), *, *p* = 0.0201. Dots represent values from individual mice, *n* = 2053–2324 cells from 2 male and 2 female mice per time point. ***I***, ***J***, Graphs display the mean ± SEM percentage of *Grp^+^* (***I***) or *Neurod6* ^+^ (***J***) DA neurons across subregions of the VTA and SNc in male (M) and female (F) mice. Unpaired *t* tests between males and females for each region revealed no significant sex differences. Dots represents values from individual mice, *n* = 3586 cells from 6 male mice and 3424 cells from 6 female mice. IF, interfascicular nucleus; PN/PIF, paranigral/parainterfascicular nuclei; PBP, parabrachial pigmented nucleus; SNc-V, substantia nigra pars compacta-ventral portion; SNc-D, substantia nigra pars compacta-dorsal portion. See also Fig. 1-1.

To define subclasses of DA neurons in an unbiased way, we performed single-cell RNA sequencing of DA neurons from P26–P34 male and female mice in which dopamine transporter (DAT)-expressing neurons were labeled with a tdTomato reporter (DAT^IRES^Cre;Ai9, Fig. 1-1*A*). A bioinformatic and statistical workflow revealed nine clusters of DA neurons based on differential gene expression (Fig. 1-1*B–E* and Table 1-1). We identified markers for each cluster and validated eight of the nine clusters. Two of the clusters corresponded to DA subpopulations in the hypothalamus, four defined subclasses of VTA neurons, and two corresponded to the SNc (Fig. 1-1*F*). These populations are consistent with recent single-cell DA neuron profiling studies (Poulin et al., 2014; La Manno et al., 2016; Romanov et al., 2017).

10.1523/ENEURO.0152-18.2018.f1-1 Figure 1-1 Single-cell RNA-sequencing defines genetic subpopulations of mouse midbrain dopamine neurons. ***A***, Confocal image of a midbrain section from a DAT^IRES^Cre;Ai9 mouse showing tdTomato Cre-reporter expression in midbrain dopamine (DA) neurons. ***B***, *t*-distributed stochastic neighborhood embedding (tSNE) plot shows genetically defined clusters of DA neurons. 232 cells were analyzed from 8 mice. ***C***, Heatmap displays a selection of differentially expressed genes in cluster 8 (highlighted by the dashed box). Individual genes are along the *Y*-axis and individual cells are along the *X*-axis. Colored bars at the top indicate the assigned cell cluster. ***D***, Heatmap of cell coclustering. The RSEC clustering method is based on consensus clustering over many parameters. To assess the robustness of the final clustering, we generated a coclustering matrix where, for each pair of cells, we recorded the proportion of times in which two cells clustered together. The cells that failed to cluster at least 50% of the time were dropped from the analysis. The plot can be used to assess the robustness of the clustering procedure. Clusters 1, 2, 3, 7, and 8 are very robust, as almost all of the cells cluster together 100% of the time. Clusters 4, 5, and 6 are less robust, meaning that their boundaries are not well defined. Both the *X*- and Y-axis correspond to cells, ordered according to hierarchical clustering; colored bars at the top indicate the final nine cluster labels. ***E***, Heatmap of the top 151 differentially expressed genes. Individual genes are along the *Y*-axis and individual cells are along the *X*-axis (n = 232 cells). Colored bars at the top indicate cell cluster and sex of the animal. Differentially expressed genes were obtained by performing all pairwise comparisons among the nine defined clusters with the R package *limma*. Genes were sorted by *p*-value, and the top 10 genes per comparison were selected for visualization (with nine clusters there are a total of 36 pairwise comparisons). If a gene was identified as differentially expressed in more than one comparison, it was reported in the heatmap only once. This procedure resulted in 151 distinct differentially expressed genes. ***F***, Table summarizing selected markers and the anatomical location of each genetically defined cluster. Anatomical location was determined by *in situ* hybridization of marker genes and expression data from the Allen Brain Atlas. Marker genes were included in the table if there was significant differential expression from at least three other clusters. Download Figure 1-1, TIF file.

10.1523/ENEURO.0152-18.2018.t1-1 Table 1-1 Differential gene expression by cluster Excel table reports gene names, expression values, and p values for the genes used to generate the heatmap in Figure 1-1E. Cluster comparison = Clusters that are compared where cluster n is expressed as Xn. Gene = differentially expressed gene between the two clusters compared in “Cluster comparison”. Log Fold Change = log-fold change of gene expression between the two clusters in “Cluster comparison”. Negative value indicates increased expression in the second cluster (e.g. a negative value in X1-X2 means the gene is higher expressed in X2 relative to X1). Average Expression = Average expression of the gene across all cells. T = moderated t-statistic from the limma DE test. P.Value = probability that the gene is differentially expressed between the two clusters. Adj.P.Val = p value adjusted for multiple testing using the Benjamini-Hochberg method. B = the log-odds that the gene is differentially expressed. Download Table 1-1, XLSX file.

### *Grp* and *Neurod6* define anatomically overlapping but distinct midbrain subpopulations

Consistent with prior studies (Poulin et al., 2014; La Manno et al., 2016), we identified a cell cluster that showed relatively high and selective expression of *Grp*, *Neurod6*, and *Gpr83* (cluster #8, Fig. 1-1*C*). Given recent interest in *Neurod6* and *Grp* as markers that label ventromedial VTA DA neurons (La Manno et al., 2016; Viereckel et al., 2016; Khan et al., 2017), we chose to quantitatively analyze their expression patterns in the midbrain and examine the extent of their overlap. Using multiplex FISH, we found that in adult mice, *Grp*-expressing neurons represented 29.9% ± 0.5% of the total midbrain DA neuron population (1804/6407 neurons from 8 mice) and 35.9% ± 0.4% of DA neurons in the VTA (1570/4377 neurons from 8 mice; Fig. 1*A–D*). 97.4% ± 0.7% of *Grp* ^+^ cells in the midbrain were dopaminergic, defined by coexpression of tyrosine hydroxylase (*Th*) mRNA (1780/1822 cells from 4 mice). *Grp* ^+^ DA neurons were found in all subregions of the VTA but were enriched in the ventromedial portions of the VTA, the interfascicular nucleus (IF), and paranigral/parainterfascicular nuclei (PN/PIF; *p* < 0.0001,^a^ one-way ANOVA with Tukey’s *post hoc* test, Fig. 1*E*). Notably, while *Grp* was previously identified as a VTA marker (Chung et al., 2005; Greene et al., 2005; Viereckel et al., 2016), we also found *Grp*-expressing DA neurons in the ventromedial portion of the SNc (22.7% ± 1.8% of DA neurons in this region, Fig. 1*A–F*).

*Neurod6* expression defined a more restricted DA population, accounting for 26.8% ± 0.5% of VTA DA neurons (1172/4377 neurons from 8 mice), with the highest density of *Neurod6* ^+^ DA neurons in the ventromedial IF and PN/PIF regions (*p* < 0.0001,^b^ one-way ANOVA with Tukey’s *post hoc* test, Fig. 1*E*). In contrast to *Grp*, *Neurod6* mRNA was not expressed in the SNc (Fig. 1*A–F*). 93.1% ± 0.6% of *Neurod6* ^+^ VTA neurons were dopaminergic (948/1016 cells from 4 mice). Consistent with our RNA-seq data, we found that most, but not all, *Neurod6^+^* DA neurons coexpressed *Grp* (77.5% ± 0.8%, 909/1172 cells from 8 mice). Broken down by subregion, the majority of *Neurod6* ^+^ DA neurons in the IF and PN/PIF coexpressed *Grp*; however, a third of *Neurod6* ^+^ DA neurons in the PBP did not have detectable expression of *Grp* (Fig. 1*F*). *Grp*-expressing but *Neurod6*-lacking DA neurons were found throughout the VTA, and nearly all of the *Grp^+^* DA neurons in the SNc were lacking *Neurod6* (Fig. 1*F*).

Together, these data show that *Grp* is expressed in a subpopulation of midbrain DA neurons spanning the VTA and ventromedial portion of the SNc. *Neurod6* is expressed in a more restricted midbrain population, defining a subgroup of the *Grp^+^* DA population, which is located exclusively in the VTA. In addition, there is a small subset of VTA DA neurons that express *Neurod6* but not *Grp*.

To determine if these cell populations were present throughout development, we assessed *Grp* and *Neurod6* expression at multiple ages. We found that while there were subtle increases in the percentages of *Grp* ^+^ VTA DA neurons from 8 to 16 wk (*p* = 0.036,^c^ one-way ANOVA with Tukey’s *post hoc* test, Fig. 1*G*) and *Neurod6* ^+^ VTA DA neurons from 2 to 16 wk (*p* = 0.024,^d^ one-way ANOVA with Tukey’s *post hoc* test, Fig. 1*H*), the proportions of DA neurons expressing these markers were largely stable from 2 wk postnatal through adulthood (P14–P112). We also investigated whether these cell populations were similar between sexes. We found that the *Grp*- and *Neurod6*-expressing DA subpopulations were present in male and female mice in similar proportions throughout VTA and SNc subregions (see statistics worksheet for unpaired *t* test *p* values,^e–m^ Fig. 1*I*,*J*). Furthermore, in the RNA-seq data, we found no significant differences in expression levels of *Grp* or *Neurod6* between males and females (log2-fold-change of 1.70 for *Neurod6, p* = 0.51^n^ and 0.94 for *Grp*, *p* = 0.68^o^ in males versus females, likelihood ratio test). These data demonstrate that the *Grp* ^+^ and *Neurod6* ^+^ DA neuron populations are present in both sexes and stable over time.

### *Grp* and *Neurod6*-expressing neurons project to the nucleus accumbens medial shell

**Figure 2.**
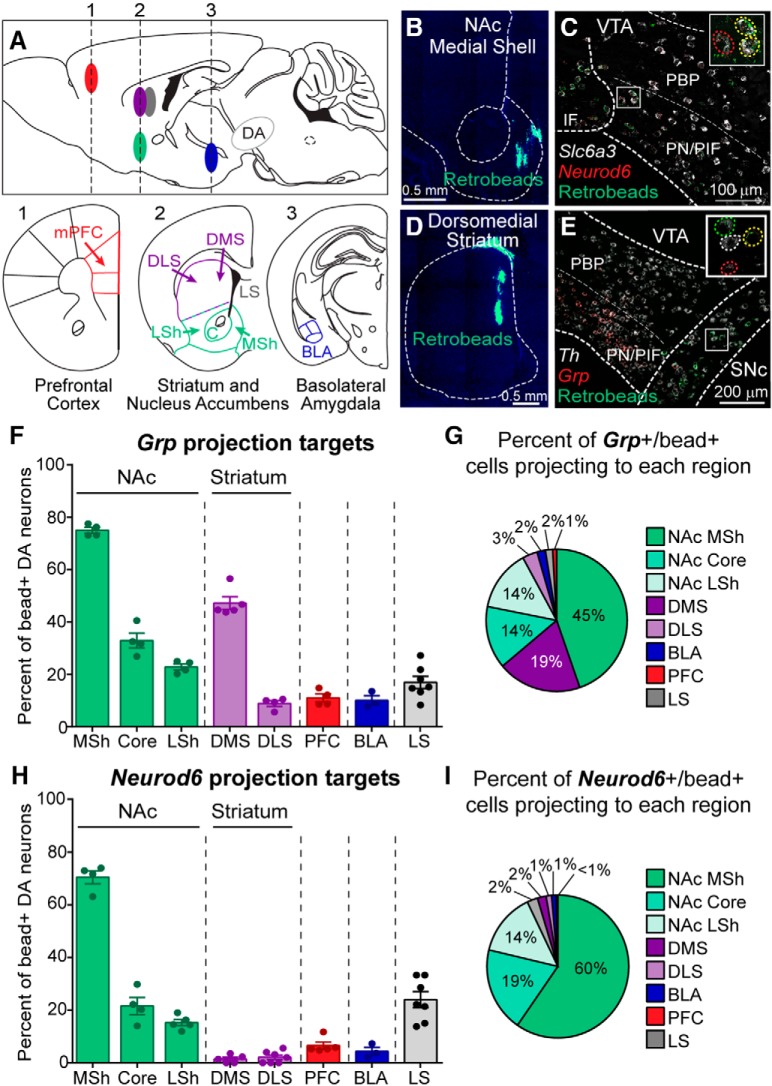
Projection targets of *Grp* ^+^ or *Neurod6^+^* DA neurons. ***A***, Schematic of retrobead injection sites in sagittal view. Numbers correspond to the coronal section schematics below showing the eight projection target sites. mPFC, medial prefrontal cortex; DLS, dorsolateral striatum; DMS, dorsomedial striatum; LSh, lateral shell; C, core; MSh, medial shell; LS, lateral septum; BLA, basolateral amygdala. ***B***, Image showing a green fluorescent retrobead injection into the nucleus accumbens (NAc) medial shell with DAPI staining in blue. ***C***, Image of the VTA showing fluorescent *in situ* hybridization (FISH) for *Slc6a3* (DAT) mRNA (white), *Neurod6* mRNA (red), and green retrobeads from a NAc medial shell injection. ***D***, Image showing a green retrobead injection into the dorsomedial striatum (DMS) with DAPI staining in blue. ***E***, FISH image of the midbrain showing *Th* mRNA (white), *Grp* mRNA (red), and green retrobeads from a DMS injection. Insets boxes in ***C*** and ***E*** show higher-magnification images of the boxed regions. Red circles identify *Neurod6^+^* (***C***) or *Grp* ^+^ (***E***) neurons, green circles define bead-positive neurons, and yellow circles show bead-positive neurons expressing *Neurod6* (***C***) or *Grp* (***E***). White circles identify neurons expressing *Th* only. ***F***, Quantification of the percentage of *Th* ^+^/bead^+^ midbrain neurons that coexpressed *Grp* mRNA for each of the projection sites. Bars represent mean ± SEM, each dot represents one mouse. ***G***, Summary of the projection targets of *Grp* ^+^ DA neurons expressed as a percentage of total bead^+^/*Grp* ^+^/*Th* ^+^ neurons. ***H***, Quantification of the percentage of DAT ^+^/bead^+^ midbrain neurons that coexpressed *Neurod6* mRNA for each of the projection sites. Bars represent mean ± SEM, each dot represents one mouse. ***I***, Summary of the projection targets of *Neurod6* ^+^ DA neurons expressed as a percentage of total bead^+^/*Neurod6* ^+^/DAT ^+^ neurons. For panels ***F–I***: NAc MSh *n* = 850 cells from 2 male and 2 female mice, NAc Core *n* = 858 cells from 2 males and 2 females, NAc LSh *n* = 1215 cells from 3 males and 1–2 females, DMS *n* = 637 cells from 3 males and 2 females, DLS *n* = 1038 cells from 2–4 males and 2–3 females, mPFC *n* = 211 cells from 3–4 males and 1 female, BLA *n* = 297 cells from 3 males, and LS *n* = 345 cells from 2 males and 4 females. See also Fig. 2-1.

VTA DA neurons comprise multiple subcircuits, which send projections to different brain regions with distinct functional consequences (Roeper, 2013). To determine if the *Grp* and *Neurod6* DA populations have specific projection targets, we combined retrograde labeling from eight primary DA neuron projection sites with *Grp* and *Neurod6* FISH (Fig. 2*A*and Fig. 2-1*A*). We found that of the DA neurons projecting to the medial shell of the nucleus accumbens (NAc), 75.0% ± 1.1% were *Grp*-positive and 70.4% ± 2.5% were *Neurod6*-positive, indicating that these markers were expressed in the majority of medial shell-projecting DA neurons (Fig. 2*B*,*C*,*F*,*H*). *Grp^+^* and *Neurod6* ^+^ DA neurons also projected to the NAc core and lateral shell but represented a smaller fraction of the neurons projecting to these regions compared to the medial shell (Fig. 2*F*,*H*). When quantified as a percentage of the total marker-positive bead-labeled DA neurons across all injection sites, the NAc medial shell was the primary target region for both *Grp* ^+^ and *Neurod6* ^+^ DA neurons (Fig. 2*G*,*I*). Together, these findings indicate a strong mesoaccumbens projection from *Neurod6^+^* and *Grp^+^* VTA DA neurons.

10.1523/ENEURO.0152-18.2018.f2-1 Figure 2-1 Retrobead injection sites and location of *Grp* ^+^ DA neurons projecting to the dorsomedial striatum or NAc medial shell. ***A***, Schematics of coronal brain sections from the indicated anterior/posterior (A/P) positions from bregma. Colored regions represent retrobead injection sites from individual mice, NAc MSh n = 2 male and 2 female mice, NAc Core n = 2 male and 2 female mice, NAc LSh n = 3 male and 2 female mice, DMS n = 3 male and 2 female mice, DLS n = 4 male and 3 female mice, mPFC n = 4 male and 1 female mouse, BLA n = 3 male mice, and LS n = 2 male and 3 female mice. ***B***, ***C***, Schematics showing the locations of *Grp* ^+^/*TH* ^+^/bead^+^ cells in the midbrain that project to the dorsomedial striatum (***B***) or nucleus accumbens (NAc) medial shell (***C***). Schematics were generated from representative single FISH images. Download Figure 2-1, TIF file.

We found that *Neurod6* mRNA was largely absent from DA neurons projecting to regions outside of the NAc, indicating a selective output of the *Neurod6^+^* DA neuron population (Fig. 2*H*,*I*). By contrast, *Grp^+^* DA neurons represented a substantial percentage (47.3% ± 2.4%) of DA neurons projecting to the dorsomedial striatum (DMS; Fig. 2D–G). 87.7% ± 2.5% of the DMS-projecting *Grp-*positive neurons were located in the ventromedial portion of the SNc (Fig. 2-1*B*), consistent with prior reports mapping dopaminergic projections to the DMS (Lerner et al., 2015). These neurons lacked expression of *Neurod6*, as essentially no DMS-projecting neurons were *Neurod6*-positive (Fig. 2*H*,*I*). Taken together, these results indicate that there are at least two populations of DA neurons that express *Grp*: those located in the VTA that project primarily to the medial NAc and those in the ventromedial SNc, which project selectively to the DMS (Fig. 2-1*B*,*C*).

A recent study showed that *Neurod6*-expressing DA neurons, labeled by a Cre reporter in NEX-Cre mice [*Neurod6* was previously referred to as *NEX* (Goebbels et al., 2006)], project to the lateral septum (Khan et al., 2017). We found relatively few lateral septum-projecting neurons in the midbrain, of which 37.4% ± 3.1% were non-dopaminergic (92/252 cells from 4 mice). Of the DA neurons projecting to the lateral septum, 17.0% ± 2.3% were *Grp^+^* and 24.0% ± 3.0% were *Neurod6* ^+^ (Fig. 2*F*,*H*). Quantified as a percentage of total bead-labeled neurons, <2% of *Grp* ^+^ or *Neurod6* ^+^ DA neurons projected to the lateral septum (Fig. 2*G*,*I*). These results indicate that compared to other brain regions, the lateral septum is not a major target for *Grp-* or *Neurod6*-expressing DA neurons.

### *Neurod6^+^* DA neurons have unique physiological properties

**Figure 3.**
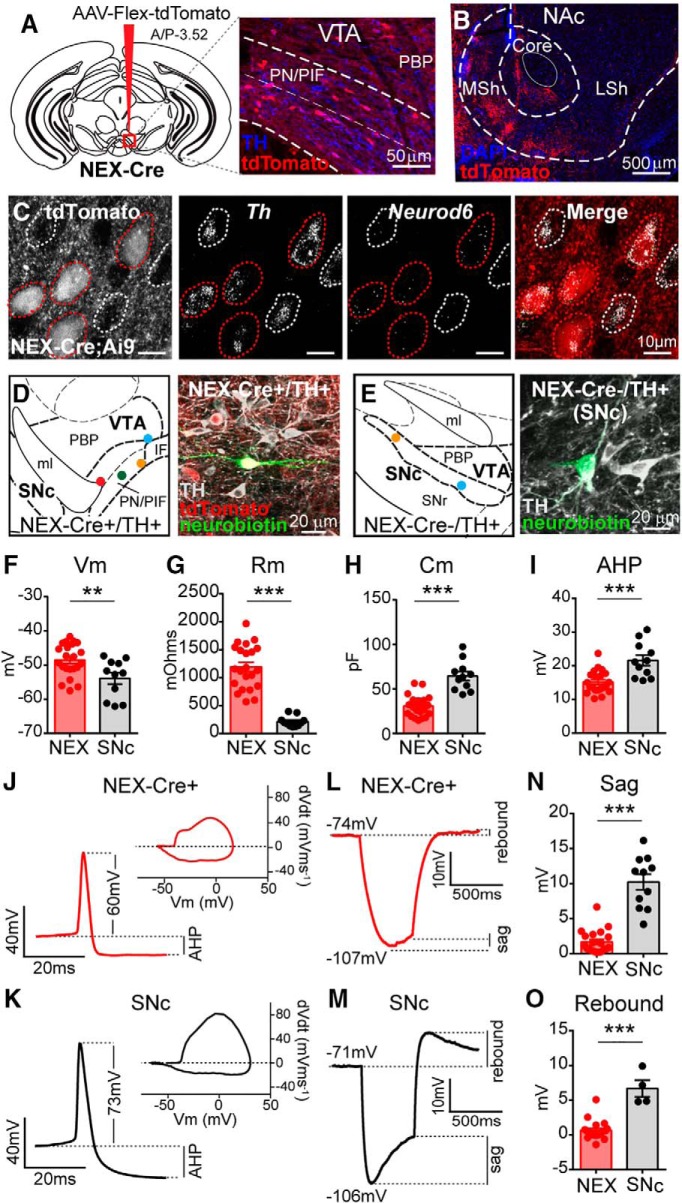
*Neurod6*-expressing DA neurons have noncanonical physiological properties. ***A***, Cre-dependent tdTomato-expressing virus (AAV-Flex-tdTomato) was injected unilaterally into the VTA of a NEX-Cre mouse. Right panel shows tdTomato-labeled NEX-Cre^+^ neurons in the ventromedial VTA. Tyrosine hydroxylase (TH) staining is in blue. ***B***, Confocal image of the nucleus accumbens (NAc) showing tdTomato-labeled projections from midbrain NEX-Cre^+^ neurons. MSh, medial shell; LSh, lateral shell. ***C***, Confocal images of VTA neurons from NEX-Cre;Ai9 mice with fluorescent *in situ* hybridization (FISH) for tyrosine hydroxylase (*Th*) and *Neurod6* mRNA. The tdTomato Cre-reporter is expressed in NEX-Cre^+^ neurons. NEX-Cre^+^/*Neurod6* ^+^ DA neurons are circled in red, NEX-Cre-/*Neurod6^+^* DA neurons are circled in white. ***D***, ***E***, Left panels show representative recording sites of NEX-Cre^+^ VTA (***D***) and NEX-Cre- SNc neurons (***E***). Circles show the locations of recorded DA neurons. Right panels show examples of recorded, neurobiotin-filled neurons positive for TH. ***F–I***, Graphs display the mean ± SEM membrane potential (***F***, Vm, **, *p* = 0.0075), membrane resistance (***G***, Rm, ***, *p* < 0.0001), capacitance (***H***, Cm, ***, *p* < 0.0001), and afterhyperpolarization (***I***, AHP, ***, *p* = 0.0002) for NEX-Cre^+^ and SNc neurons. ***J***, ***K***, Representative action potential traces and phase plots (mVms^−1^/mV) from a NEX-Cre^+^ (***J***) and SNc (***K***) neuron. ***L***, ***M***, Representative responses to a 500 ms negative current step from a NEX-Cre^+^ (–25 pA, ***L***) and SNc (–150 pA, ***M***) neuron. ***N***, Quantification of the mean ± SEM sag component in NEX-Cre^+^ and SNc neurons (***, *p* < 0.0001). ***O***, Quantification of the mean ± SEM rebound depolarization from neurons hyperpolarized to –100 ± 7 mV (***, *p* < 0.0001). An unpaired *t test* was used for all comparisons. For all panels, dots represent values from individual cells, NEX-Cre^+^ *n* = 7 male and 7 female mice, NEX-Cre-/SNc *n* = 4 male mice and 1 female mouse. See Fig. 3-1 for a complete summary of the electrophysiology results.

DA neurons projecting to different target areas possess distinct electrophysiological profiles (Lammel et al., 2008; Margolis et al., 2008; Lerner et al., 2015). To investigate whether *Neurod6* expression defines a physiologically distinct subclass of DA neurons, we used NEX-Cre knock-in mice (Goebbels et al., 2006). We injected virus expressing a Cre-dependent tdTomato reporter (AAV-Flex-tdTomato) into the midbrain and found NEX-Cre^+^ DA neurons along the ventromedial portion of the VTA (Fig. 3*A*), consistent with the expression pattern of *Neurod6* mRNA. In agreement with our tracing data, we found that tdTomato-labeled NEX-Cre^+^ neurons projected to the NAc medial shell and core (Fig. 3*B*). To visualize NEX-Cre^+^ neurons for physiology, we crossed NEX-Cre mice to the Ai9 tdTomato Cre-reporter mouse line (Madisen et al., 2010). We performed FISH for *Neurod6* mRNA in NEX-Cre;Ai9 mice (Fig. 3*C*) and found that 93.6% ± 1.2% of tdTomato–positive DA neurons in the VTA coexpressed *Neurod6* (331/353 cells from 4 mice), making this a suitable model to use for targeted electrophysiology recordings. Consistent with the 77.5% of *Neurod6* ^+^ neurons that coexpressed *Grp*, 73.7% ± 2.9% of the NEX-Cre;tdTomato–positive VTA DA neurons expressed *Grp* (175/232 cells from 4 mice). We did observe that not all *Neurod6* ^+^ VTA DA neurons expressed tdTomato in the NEX-Cre;Ai9 mice (30.4% ± 1.7% of *Neurod6* ^+^ DA neurons were tdTomato-positive, 361/1092 cells from 4 mice). In addition, a third of the tdTomato-positive midbrain neurons were non-dopaminergic (36.9% ± 3.4% *Th* negative, 193/546 cells from 4 mice). This discrepancy may be due to transient Cre expression in non-DA neurons during development. These data suggest that NEX-Cre mice may not be appropriate for studies requiring selective access to the entire *Neurod6* ^+^ VTA DA subpopulation, but can be used to target *Neurod6* ^+^ cells for whole-cell recordings in which dopaminergic identity can be confirmed *post hoc*.

10.1523/ENEURO.0152-18.2018.f3-1 Figure 3-1 Summary of electrophysiology data. Extended Data table supporting Fig. 3. Table reports sample size, group means ± SEM, and unpaired *t*-test *p* values for each parameter measured. M, male mice; F, female mice. Download Figure 3-1, DOCX file.

To determine if *Neurod6*-expressing neurons represent a functionally distinct cell class, we recorded from tdTomato-labeled NEX-Cre^+^ neurons in the VTA and analyzed their intrinsic membrane properties, action potential waveform, and response to hyperpolarizing current injection (see Fig. 3-1 for a summary of the physiology data including sample sizes). We confirmed that the NEX-Cre^+^ neurons analyzed were dopaminergic by filling patched neurons with neurobiotin and costaining for TH (Fig. 3*D*,*E*). We found that NEX-Cre^+^ DA neurons had a distinct electrophysiological signature compared to “classic” SNc DA neurons. Specifically, NEX-Cre^+^ DA neurons exhibited a more depolarized membrane potential (Vm, *p* = 0.0075,^p^ unpaired *t* test), had higher membrane resistance (Rm, *p <* 0.0001,^q^ unpaired *t* test), and reduced membrane capacitance (Cm, *p* < 0.0001,^r^ unpaired *t* test) compared to SNc DA neurons (Fig. 3*F–H*). The action potential height of NEX-Cre^+^ DA neurons was also significantly shorter (*p* < 0.0001,^s^ unpaired *t* test), and they had a less pronounced afterhyperpolarization (AHP, *p* = 0.0002,^t^ unpaired *t* test; Fig. 3*I–K*). Due to their high membrane resistance, NEX-Cre^+^ DA neurons required less negative current to hyperpolarize to –100 mV compared to SNc DA neurons (–25 to –50 pA for NEX-Cre^+^ neurons versus –150 pA for SNc neurons). NEX-Cre^+^ DA neurons had a significantly smaller sag component, which is indicative of smaller I_h_ (*p* < 0.0001,^u^ unpaired *t* test, Fig. 3*L–N*) and less rebound depolarization (*p* < 0.0001,^v^ unpaired *t* test, Fig. 3*L*,*M*,*O*). The noncanonical electrophysiological characteristics of NEX-Cre^+^ neurons are consistent with those reported for NAc medial shell-projecting DA neurons (Lammel et al., 2008), suggesting that *Neurod6* is a useful marker for this VTA subpopulation.

### *Neurod6*-lacking VTA DA neurons show increased susceptibility to degeneration in a 6-OHDA mouse model

**Figure 4.**
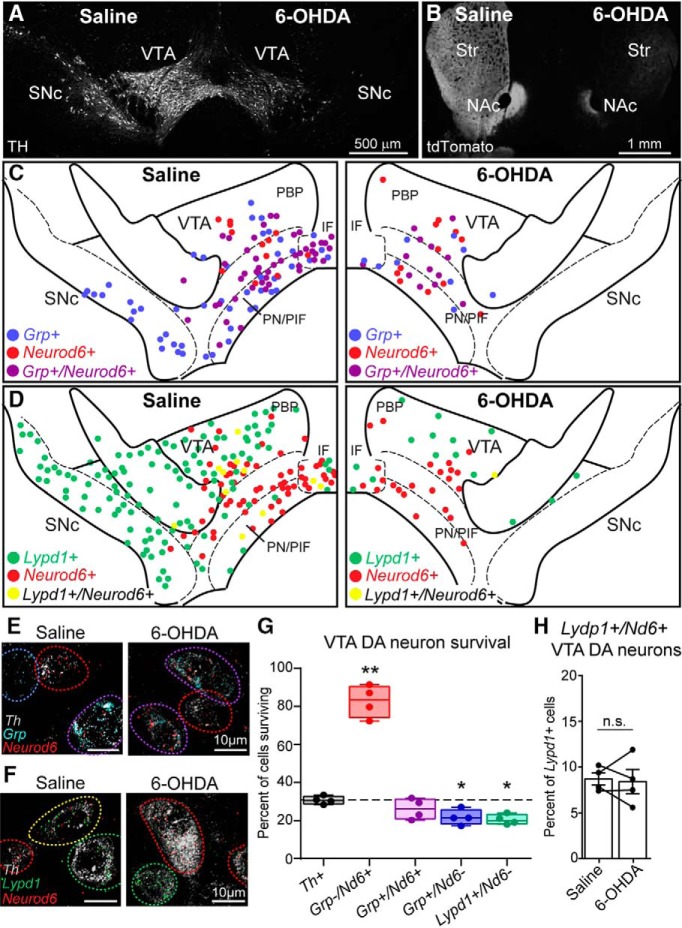
*Neurod6*-expression affects susceptibility to 6-OHDA–induced degeneration. ***A***, Confocal image of a midbrain section stained with a tyrosine hydroxylase (TH) antibody 4 wk after unilateral saline (left) or 6-OHDA (right) injection into the medial forebrain bundle. ***B***, Image of a striatal (Str) section showing loss of tdTomato^+^ axon terminals 4 wk following unilateral 6-OHDA injection in a DAT^IRES^Cre;Ai9 mouse. NAc, nucleus accumbens. ***C***, Schematic showing the location of DA neurons in the saline and 6-OHDA injected hemispheres labeled with different markers by fluorescent *in situ* hybridization (FISH): blue circles are *Grp^+^*, red circles are *Neurod6^+^*, and purple circles are *Grp^+^/Neurod6^+^*. ***D***, Schematic showing the location of *Lypd1^+^* (green), *Neurod6^+^* (red), and *Lypd1^+^/Neurod6^+^* (yellow) DA neurons in the midbrain following saline and 6-OHDA injection. ***E***, ***F***, Confocal images of FISH for the indicated markers. ***E***, Images show *Grp^+^* (blue circle), *Neurod6^+^* (red circles), and *Grp^+^/Neurod6^+^* (purple circles) DA neurons in the VTA following saline and 6-OHDA injection. ***F***, Images show *Lypd1^+^* (green circles), *Neurod6^+^* (red circles), and *Lypd1^+^/Neurod6^+^* (yellow circle) DA neurons in the VTA following saline and 6-OHDA injection. ***G***, Box-and-whisker plots (min to max) show the percentage of VTA DAT neurons expressing each set of markers in the 6-OHDA–injected hemisphere compared to the saline-injected hemisphere. Dotted line represents the percentage of all VTA DA neurons (*Th^+^*) surviving in the 6-OHDA hemisphere. Dots represent data from individual mice, *n* = 4 female mice. A paired one-way ANOVA with Dunnett’s multiple comparisons test was used to compare each subpopulation to all *Th^+^* VTA DA neurons (*n* = 4376 saline and 1352 6-OHDA cells): *Grp^–^/Neurod6^+^* (*n* = 184 saline and 151 6-OHDA cells), ** p = 0.0022; *Grp^+^/Neurod6^+^* (*n* = 763 saline and 207 6-OHDA cells), *p* = 0.3169; *Grp^+^/Neurod6^–^* (*n* = 749 saline and 165 6-OHDA cells), * *p* = 0.0246; *Lypd1^+^/Neurod6^–^* (*n* = 1056 saline and 171 6-OHDA cells), * *p* = 0.0313; *Nd6* = *Neurod6*. ***H***, Bar graphs display the percentage of *Lypd1* ^+^ VTA DA neurons that coexpress *Neurod6* mRNA in the saline-injected (*n* = 102/3750 cells) and 6-OHDA–injected (*n* = 16/904 cells) hemispheres. Bars represent mean ± SEM, dots represent the values from individual mice, *n* = 4 female mice; paired *t* test, n.s., not significant, *p* = 0.8091.

In addition to their anatomic location, projection targets, and physiology, vulnerability to degeneration is a key feature of DA neurons that differs by subtype. Previous *in vitro* studies have implicated both *Neurod6* and *Grp* as being potentially neuroprotective (Chung et al., 2005; Uittenbogaard and Chiaramello, 2005; Uittenbogaard et al., 2010; Baxter et al., 2012). In mice, expression of *Neurod6* and the related transcription factor *Neurod1* are important for survival of DA neurons during development (Khan et al., 2017). *Grp*-expressing cells have been observed in postmortem tissue from PD patients, suggesting that *Grp* expression may be related to cell survival (Viereckel et al., 2016). We therefore investigated the sensitivity of *Neurod6* and/or *Grp*-expressing VTA DA neurons to degeneration in a mouse model of PD. To do this, we injected the dopaminergic toxin 6-hydroxydopamine (6-OHDA) unilaterally into the medial forebrain bundle of adult (P120) female mice (Thiele et al., 2012). 6-OHDA resulted in a progressive, unilateral loss of DA neurons with a 96.0% ± 0.6% reduction in SNc DA neurons and a 69.3 ± 1.1% loss of VTA DA neurons after 4 wk (Fig. 4*A*). We confirmed DA axon denervation throughout the striatum in the 6-OHDA–injected hemisphere, with notable sparing of DA projections to the NAc medial shell and core (Fig. 4*B*). DA neuron loss led to unilateral motor impairment as measured by the cylinder test (*p* = 0.0002,^w^ one-way ANOVA with Tukey’s *post hoc* test: saline paw vs. 6-OHDA paw *p* = 0.0163, saline paw vs. both paws *p* = 0.0157, 6-OHDA paw vs. both paws *p* = 0.0002, *n* = 4 mice).

To determine how *Grp*- and *Neurod6*-expressing DA neurons in the VTA responded to 6-OHDA (Fig. 4*C–F*), we compared the reduction of *Grp* ^+^ and/or *Neurod6* ^+^ VTA DA neurons between the saline and 6-OHDA hemisphere to all VTA DA neurons defined by *Th* expression (Fig. 4*G*). For this analysis, we included only VTA DA neurons, as essentially all SNc neurons (including *Grp^+^* SNc neurons) degenerated in the 6-OHDA–injected hemisphere (Fig. 4*A*,*C*,*D*). We found that 4 wk following 6-OHDA injection, 26.1% ± 2.7% of *Grp* ^+^*/Neurod6^+^* DA neurons survived, which was similar to the percentage of total *Th* ^+^ VTA neurons surviving (30.7% ± 1.1%; *p* < 0.0001,^x^ paired one-way ANOVA; *p* = 0.3169, Tukey’s *post hoc* test; Fig. 4*G*). Notably, VTA DA neurons that expressed only *Neurod6* and not *Grp* (*Grp-/Neurod6^+^*) were significantly spared compared to the rest of VTA DA neurons, with 82.7% ± 4.2% of neurons surviving (*p* = 0.0022, Tukey’s *post hoc* test, Fig. 4*G*). By contrast, VTA DA neurons that expressed *Grp* but not *Neurod6* (*Grp^+^/Neurod6* ^–^) showed slightly increased vulnerability compared to all VTA DA neurons, with 21.7% ± 2.0% surviving (*p =* 0.0246, Tukey’s *post hoc* test, Fig. 4*G*). Therefore, the DA neuron subpopulations marked by expression of *Grp* and/or *Neurod6* have different responses to 6-OHDA, and VTA neurons lacking *Neurod6* are more susceptible to degeneration.

An increase in the proportion of *Neurod6^+^* DA neurons in the VTA following 6-OHDA could be due to selective sparing of the *Neurod6* cell population or from *Neurod6* expression turning on in surviving neurons of other VTA subpopulations. To attempt to distinguish these possibilities, we performed FISH for another VTA DA neuron subtype marker *Lypd1* (La Manno et al., 2016).

Under normal conditions, *Lypd1* was expressed almost exclusively in the PBP subregion of the VTA as well as throughout the SNc (Fig. 4*D*,*F*). *Neurod6* was generally not coexpressed in *Lypd1^+^* DA neurons (7.0% ± 1.6% of *Lypd1^+^* DA neurons coexpressed *Neurod6*, Fig. 4*D*,*F*,*H*), indicating that these markers define distinct cell populations. In response to 6-OHDA, we found that 20.4% ± 1.3% of *Lypd1^+^/Neurod6*- VTA DA neurons survived, which was significantly lower than the total VTA DA population (*p* = 0.0403, Tukey’s *post hoc* test, Fig. 4*G*). The surviving *Lypd1* ^+^ VTA DA neurons did not turn on expression of *Neurod6*, as the proportion of *Neurod6^+^/Lypd1^+^* VTA DA neurons remained low and was the same between the saline-injected and 6-OHDA–injected hemispheres (*p* = 0.8091,^y^ paired *t* test, Fig. 4*H*). These results suggest that the VTA DA subpopulation defined by *Neurod6* expression, which lacks *Grp* or *Lypd1*, is preferentially spared in response to a neurotoxic challenge.

## Discussion

Midbrain DA neurons are small in number but vast in their behavioral influence (Björklund and Dunnett, 2007; Roeper, 2013). As such, dopaminergic dysfunction is associated with numerous psychiatric and neurologic disorders ranging from drug addiction to PD (Damier et al., 1999; Alberico et al., 2015; Nutt et al., 2015). Recent studies have revealed that the dopaminergic system is heterogeneous at multiple levels from gene expression to circuitry to physiology to behavior (Roeper, 2013; Morales and Margolis, 2017). To tackle this heterogeneity, genetic markers that define functional DA neuron subtypes are needed. This would enable the generation of tools that allow selective manipulation of dopaminergic subpopulations. Here, we investigated two markers that we and others have identified as labeling dopaminergic subpopulations, *Grp* and *Neurod6*. We show that the combinatorial expression of these genes defines the anatomic location, projection target, physiology, and disease susceptibility of DA neurons.

We found that *Grp*, which encodes the neuropeptide gastrin-releasing peptide (Roesler and Schwartsmann, 2012), was expressed in a third of midbrain DA neurons, of which more than half coexpressed *Neurod6.* These *Grp* ^+^/*Neurod6* ^+^ neurons resided in the VTA and projected to the medial portions of the NAc. This is consistent with prior reports showing that *Grp* is expressed in a subpopulation of VTA DA neurons that shows overlap with *Neurod6*-expressing neurons (Chung et al., 2005; Greene et al., 2005; Poulin et al., 2014; La Manno et al., 2016; Viereckel et al., 2016). The fact that these neurons project to the NAc corroborates a projection-specific translational profiling study reporting that ribosome-bound *Grp* mRNA was enriched in the population of VTA DA neurons projecting to the NAc (Ekstrand et al., 2014). Notably, we also identified a previously undiscovered population of *Grp* ^+^ DA neurons that lack *Neurod6*, which were located in the ventromedial portion of the SNc. These neurons sent projections to the DMS with very little innervation of the DLS. The anatomic location of these cells in the ventral SNc is consistent with DA neurons that project to the DMS, which have unique physiological and behavioral properties compared to DLS-projecting DA neurons located in the dorsal SNc (Lerner et al., 2015).

*Neurod6* expression defined a smaller population of DA neurons that were located in the ventromedial VTA, projected selectively to the NAc, and exhibited noncanonical physiologic properties. While *Neurod6* has previously been identified as a VTA marker (Chung et al., 2005; La Manno et al., 2016; Viereckel et al., 2016; Khan et al., 2017), our study is the first to systematically map the projection sites of these cells, revealing a strong medial shell NAc projection with very little output to other DA target regions. This medial shell NAc projection is consistent with the physiology of *Neurod6* ^+^ DA neurons, which showed unique characteristics similar to those previously reported for medial NAc-projecting DA neurons defined by retrograde labeling (Lammel et al., 2008). Mesoaccumbens projections are important for mediating reward-seeking behaviors (Yun et al., 2004; Ikemoto, 2007; Lammel et al., 2011, 2014). Therefore, our identification of *Neurod6* as a marker for this cell population enables future mechanistic investigations into how this DA subcircuit controls motivated behaviors, the dysfunction of which may be important for the pathophysiology of psychiatric disorders such as drug addiction.

It was previously reported that *Neurod6* ^+^ neurons project to the lateral septum based on the axon projections of NEX-Cre mice and fluorogold retrograde labeling (Khan et al., 2017). However, projections from the VTA to the lateral septum are relatively sparse compared to the striatum and NAc (Beckstead et al., 1979; Swanson, 1982; Beier et al., 2015). To investigate this further, we performed retrobead injections into the dorsal and intermediate regions of the lateral septum and found that relatively few VTA DA neurons projected to this area. The lateral septum therefore represented only 2% of total bead-labeled *Neurod6* ^+^ DA cells across all injection sites. This indicates that under our conditions, the lateral septum was not a primary projection site of *Neurod6* ^+^ DA neurons. One possible reason for the discrepancy is that the Khan et al. (2017) study relied exclusively on the NEX-Cre mouse line, as opposed to endogenous *Neurod6* expression as done here. We found that a substantial proportion (37%) of the VTA neurons labeled in NEX-Cre mice are non-dopaminergic and that many of the VTA neurons projecting to the lateral septum are also non-dopaminergic (37%), which may have contributed to the differing results.

A defining feature of DA neurons is their susceptibility or resilience to degeneration in the context of PD (Damier et al., 1999; Alberico et al., 2015). As such, significant effort has been made to identify the molecules that determine vulnerability, as this has clear clinical importance (Brichta and Greengard, 2014). Interestingly, in addition to being genes that define specific DA neuron subtypes, both *Neurod6* and *Grp* have been shown to confer neuroprotection in cell culture models (Chung et al., 2005; Uittenbogaard and Chiaramello, 2005; Uittenbogaard et al., 2010; Baxter et al., 2012). To determine if the neuronal populations defined by *Neurod6* and/or *Grp* are preferentially spared in the context of PD, we performed unilateral 6-OHDA injections and compared the relative abundance of these markers in the 6-OHDA versus control hemisphere. We found that expression of either of these genes alone was not sufficient to confer neuroprotection, as *Grp* ^+^ neurons in the SNc degenerated completely and *Grp* ^+^ neurons in the VTA showed either similar (*Grp^+^/Neurod6^+^*) or greater (*Grp^+^/Neurod6-*) susceptibility relative to all VTA DA neurons. Notably, we did find that the small population of *Neurod6* ^+^ VTA DA neurons that lack *Grp* was significantly spared compared to neighboring DA neurons. This finding is consistent with a potential neuroprotective effect of *Neurod6* but indicates that other factors are likely involved, since *Neurod6* ^+^ VTA DA neurons that coexpressed *Grp* were not preferentially spared. Together, these results refine our understanding of the genetic factors contributing to vulnerable and spared DA cell types and suggest that the combinatorial expression of genes in a given cell population is important for defining vulnerability.

In summary, our work provides in-depth characterization of *Neurod6* and *Grp* expression in the midbrain and reveals previously unappreciated complexity in how these markers define specific DA subpopulations. Our results provide new insights into the genetic and functional heterogeneity of the DA system, which is just beginning to be unraveled. Future studies can use this information to design intersectional genetic tools based on the expression of two or more genes that will allow access to specific dopaminergic subpopulations. These types of tools represent powerful approaches to dissecting the complex ways in which the DA system contributes to behavior and disease.

10.1523/ENEURO.0152-18.2018.ed1 Extended Data 1 Computer code for single cell RNA-sequencing analysis. Download Extended Data 1, TXT file.

## Synthesis

Reviewing Editor: Trevor Drew, University of Montreal Decisions are customarily a result of the Reviewing Editor and the peer reviewers coming together and discussing their recommendations until a consensus is reached. When revisions are invited, a fact-based synthesis statement explaining their decision and outlining what is needed to prepare a revision will be listed below. The following reviewer(s) agreed to reveal their identity: Mary Kay Lobo, Ralph Di Leone. Note: If this manuscript was transferred from JNeurosci and a decision was made to accept the manuscript without peer review, a brief statement to this effect will instead be what is listed below.

Overall this is a nice study that improves understanding of the genetic markers that define dopamine neuron subpopulations. The authors have addressed all previous concerns.
