# Supplementary figures and images for: Combinatorial Expression of Grp and Neurod6 Defines Dopamine Neuron Populations with Distinct Projection Patterns and Disease Vulnerability
Source: eNeuro. 2018 Jun 13;5(3):ENEURO.0152-18.2018. doi: 10.1523/ENEURO.0152-18.2018 (PMC6104179; doi:10.1523/ENEURO.0152-18.2018)

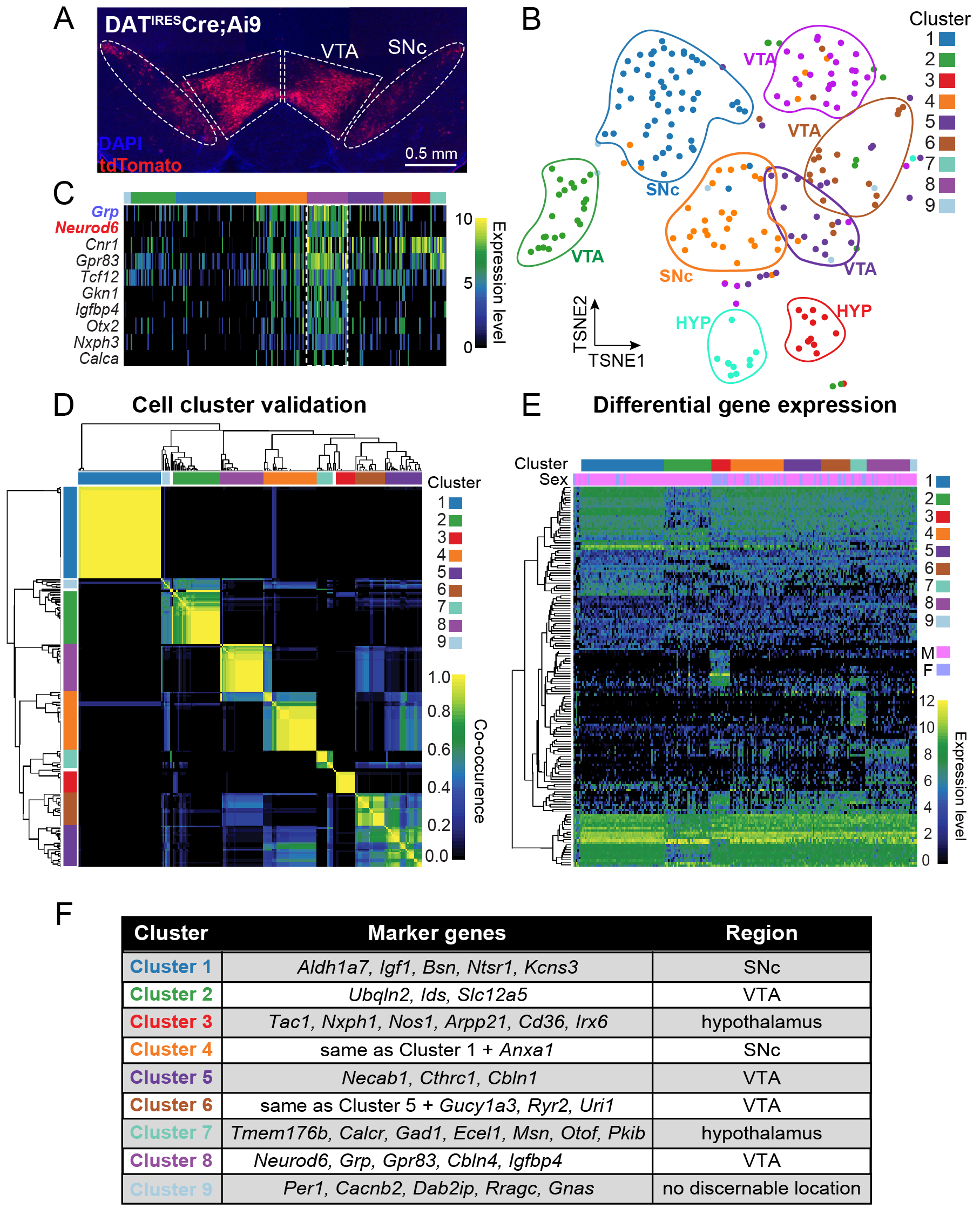

Supplement: Figure 1-1 — Single-cell RNA-sequencing defines genetic subpopulations of mouse midbrain dopamine neurons. A, Confocal image of a midbrain section from a DATIRESCre;Ai9 mouse showing tdTomato Cre-reporter expression in midbrain dopamine (DA) neurons. B, t-distributed stochastic neighborhood embedding (tSNE) plot shows genetically defined clusters of DA neurons. 232 cells were analyzed from 8 mice. C, Heatmap displays a selection of differentially expressed genes in cluster 8 (highlighted by the dashed box). Individual genes are along the Y-axis and individual cells are along the X-axis. Colored bars at the top indicate the assigned cell cluster. D, Heatmap of cell coclustering. The RSEC clustering method is based on consensus clustering over many parameters. To assess the robustness of the final clustering, we generated a coclustering matrix where, for each pair of cells, we recorded the proportion of times in which two cells clustered together. The cells that failed to cluster at least 50% of the time were dropped from the analysis. The plot can be used to assess the robustness of the clustering procedure. Clusters 1, 2, 3, 7, and 8 are very robust, as almost all of the cells cluster together 100% of the time. Clusters 4, 5, and 6 are less robust, meaning that their boundaries are not well defined. Both the X- and Y-axis correspond to cells, ordered according to hierarchical clustering; colored bars at the top indicate the final nine cluster labels. E, Heatmap of the top 151 differentially expressed genes. Individual genes are along the Y-axis and individual cells are along the X-axis (n = 232 cells). Colored bars at the top indicate cell cluster and sex of the animal. Differentially expressed genes were obtained by performing all pairwise comparisons among the nine defined clusters with the R package limma. Genes were sorted by p-value, and the top 10 genes per comparison were selected for visualization (with nine clusters there are a total of 36 pairwise comparisons). If a ge [file sup_enu-eN-NWR-0152-18-s01.tif]

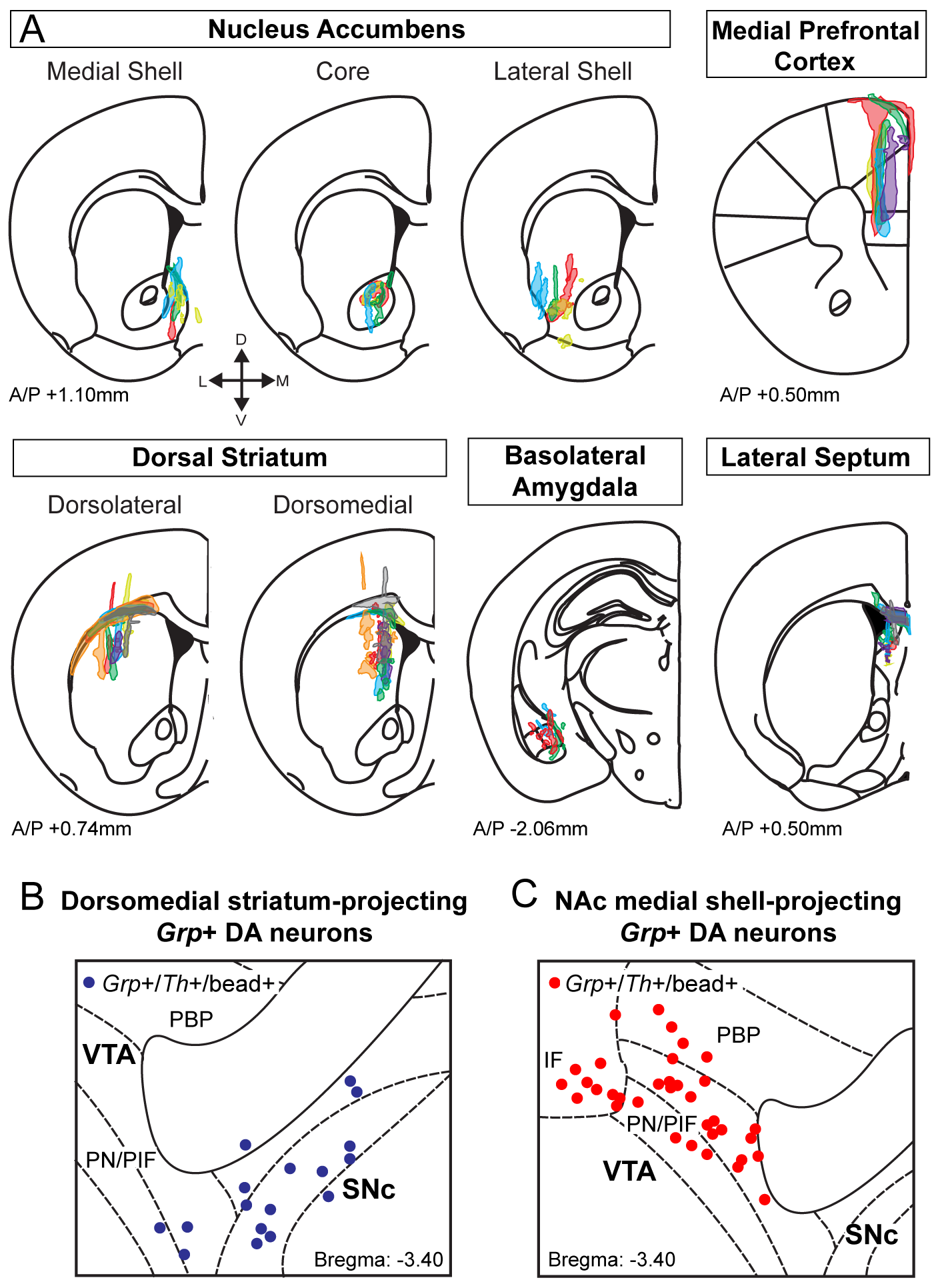

Supplement: Figure 2-1 — Retrobead injection sites and location of Grp + DA neurons projecting to the dorsomedial striatum or NAc medial shell. A, Schematics of coronal brain sections from the indicated anterior/posterior (A/P) positions from bregma. Colored regions represent retrobead injection sites from individual mice, NAc MSh n = 2 male and 2 female mice, NAc Core n = 2 male and 2 female mice, NAc LSh n = 3 male and 2 female mice, DMS n = 3 male and 2 female mice, DLS n = 4 male and 3 female mice, mPFC n = 4 male and 1 female mouse, BLA n = 3 male mice, and LS n = 2 male and 3 female mice. B, C, Schematics showing the locations of Grp +/TH +/bead+ cells in the midbrain that project to the dorsomedial striatum (B) or nucleus accumbens (NAc) medial shell (C). Schematics were generated from representative single FISH images. Download Figure 2-1, TIF file. [file sup_enu-eN-NWR-0152-18-s02.tif]
