# Supplementary material for: Combinatorial Expression of Grp and Neurod6 Defines Dopamine Neuron Populations with Distinct Projection Patterns and Disease Vulnerability
Source: eNeuro. 2018 Jun 13;5(3):ENEURO.0152-18.2018. doi: 10.1523/ENEURO.0152-18.2018 (PMC6104179; doi:10.1523/ENEURO.0152-18.2018)
Supplement: Figure 3-1 — Summary of electrophysiology data. Extended Data table supporting Fig. 3. Table reports sample size, group means ± SEM, and unpaired t-test p values for each parameter measured. M, male mice; F, female mice. Download Figure 3-1, DOCX file. [file sup_enu-eN-NWR-0152-18-s05.docx]

# Figure 3-1

|  | **Nex-Cre+** | | | | **Nex-Cre- (SNc)** | | | |  |
| --- | --- | --- | --- | --- | --- | --- | --- | --- | --- |
| **Property** | **Mean** | **SEM** | **n**  **(cells)** | **n**  **(mice)** | **Mean** | **SEM** | **n**  **(cells)** | **n**  **(mice)** | ***p* value** |
| **Holding current at -70mV**  (pA) | -15.21 | 2.09 | 22 | 7 M  7 F | -134.5 | 18.68 | 11 | 4 M  1 F | **<0.0001** |
| **Series resistance**  (mOhms) | 13.46 | 1.48 | 22 | 7 M  7 F | 11.53 | 1.69 | 11 | 4 M  1 F | 0.4313 |
| **Membrane resistance**  (mOhms) | 1189 | 83.59 | 22 | 7 M  7 F | 213.4 | 28.47 | 11 | 4 M  1 F | **<0.0001** |
| **Membrane capacitance**  (pF) | 30.66 | 2.479 | 22 | 7 M  7 F | 64.49 | 5.05 | 11 | 4 M  1 F | **<0.0001** |
| **Resting membrane potential** (mV) | -48.54 | 1.01 | 22 | 7 M  7 F | -53.88 | 1.71 | 11 | 4 M  1 F | **0.0075** |
| **Action potential threshold**  (mV) | -33.58 | 1.31 | 21 | 7 M  7 F | -30.0 | 1.41 | 11 | 4 M  1 F | 0.0956 |
| **Action potential width at threshold** (ms) | 5.04 | 0.54 | 21 | 7 M  7 F | 3.64 | 0.35 | 11 | 4 M  1 F | 0.0895 |
| **Action potential peak**  (maximum membrane  potential, mV) | 21.95 | 1.17 | 21 | 7 M  7 F | 31.55 | 1.55 | 11 | 4 M  1 F | **<0.0001** |
| **Action potential height** (change in membrane  potential from the start of the spike to maximum depolarization, mV) | 62.58 | 1.5 | 21 | 7 M  7 F | 79.17 | 2.16 | 11 | 4 M  1 F | **<0.0001** |
| **Afterhyperpolarization** (minimum membrane  potential, mV) | -55.76 | 1.08 | 21 | 7 M  7 F | -59.02 | 2.17 | 11 | 4 M  1 F | 0.1407 |
| **Afterhyperpolarization** (change in membrane  potential from the start of the spike to maximum hyperpolarization, mV) | 15.13 | 0.72 | 21 | 7 M  7 F | 21.58 | 1.57 | 11 | 4 M  1 F | **0.0002** |
| **Sag component when**  **hyperpolarized to -100 +/-**  **7mV** (mV) | 1.7 | 0.37 | 20 | 7 M  7 F | 10.22 | 1.12 | 11 | 4 M  1 F | **<0.0001** |
| **Rebound depolarization when hyperpolarized to -**  **100 +/- 7mV** (mV) | 0.63 | 0.3 | 20 | 7 M  7 F | 6.67 | 1.21 | 4 | 2 M | **<0.0001** |
